# Exposure to environmental microbiota explains persistent abdominal pain and irritable bowel syndrome after a major flood

**DOI:** 10.1186/s13099-017-0224-7

**Published:** 2017-12-14

**Authors:** NurFadhilah Yusof, Nurhazwani Hamid, Zheng Feei Ma, Rona Marie Lawenko, Wan Mohd Zahiruddin Wan Mohammad, Deirdre A. Collins, Min Tze Liong, Toshitaka Odamaki, Jinzhong Xiao, Yeong Yeh Lee

**Affiliations:** 1 School of Medical Sciences, Universiti Sains Malaysia, Kota Bharu, Kelantan Malaysia; 2 Department of Public Health, Xi’an Jiaotong-Liverpool University, Suzhou, China; 3 De La Salle Health Sciences Institute, Dasmarinas, Cavite Philippines; 4 School of Medical and Health Sciences, Edith Cowan University, Perth, Australia; 5 School of Industrial Technology, Universiti Sains Malaysia, Gelugor, Penang Malaysia; 6 Next Generation Science Institute, Morinaga Milk Industry Co., Ltd., Tokyo, Japan

**Keywords:** Abdominal pain, Dysbiosis, Flood, Malaysia, Sanitation and hygiene practices, Small intestinal bacterial overgrowth, Water

## Abstract

**Background:**

After an environmental disaster, the affected community is at increased risk for persistent abdominal pain but mechanisms are unclear. Therefore, our study aimed to determine association between abdominal pain and poor water, sanitation and hygiene (WaSH) practices, and if small intestinal bacterial overgrowth (SIBO) and/or gut dysbiosis explain IBS, impaired quality of life (QOL), anxiety and/or depression after a major flood.

**Results:**

New onset abdominal pain, IBS based on the Rome III criteria, WaSH practices, QOL, anxiety and/or depression, SIBO (hydrogen breath testing) and stools for metagenomic sequencing were assessed in flood victims. Of 211 participants, 37.9% (*n* = 80) had abdominal pain and 17% (*n* = 36) with IBS subtyped diarrhea and/or mixed type (*n* = 27 or 12.8%) being the most common. Poor WaSH practices and impaired quality of life during flood were significantly associated with IBS. Using linear discriminant analysis effect size method, gut dysbiosis was observed in those with anxiety (Bacteroidetes and Proteobacteria, effect size 4.8), abdominal pain (Fusobacteria*, Staphylococcus, Megamonas* and *Plesiomonas*, effect size 4.0) and IBS (*Plesiomonas* and *Trabulsiella*, effect size 3.0).

**Conclusion:**

Disturbed gut microbiota because of environmentally-derived organisms may explain persistent abdominal pain and IBS after a major environmental disaster in the presence of poor WaSH practices.

**Electronic supplementary material:**

The online version of this article (10.1186/s13099-017-0224-7) contains supplementary material, which is available to authorized users.

## Background

Communicable diseases pose significant public health risks after floods, and affect millions of people worldwide [1]. Besides financial loss and psychological trauma, flood victims endure increased risks from water-borne communicable diseases especially leptospirosis and typhoid [2]. Children are most affected by diarrhoeal diseases but symptoms and psychological morbidity may be worse among adults.

**Fig. 1:**
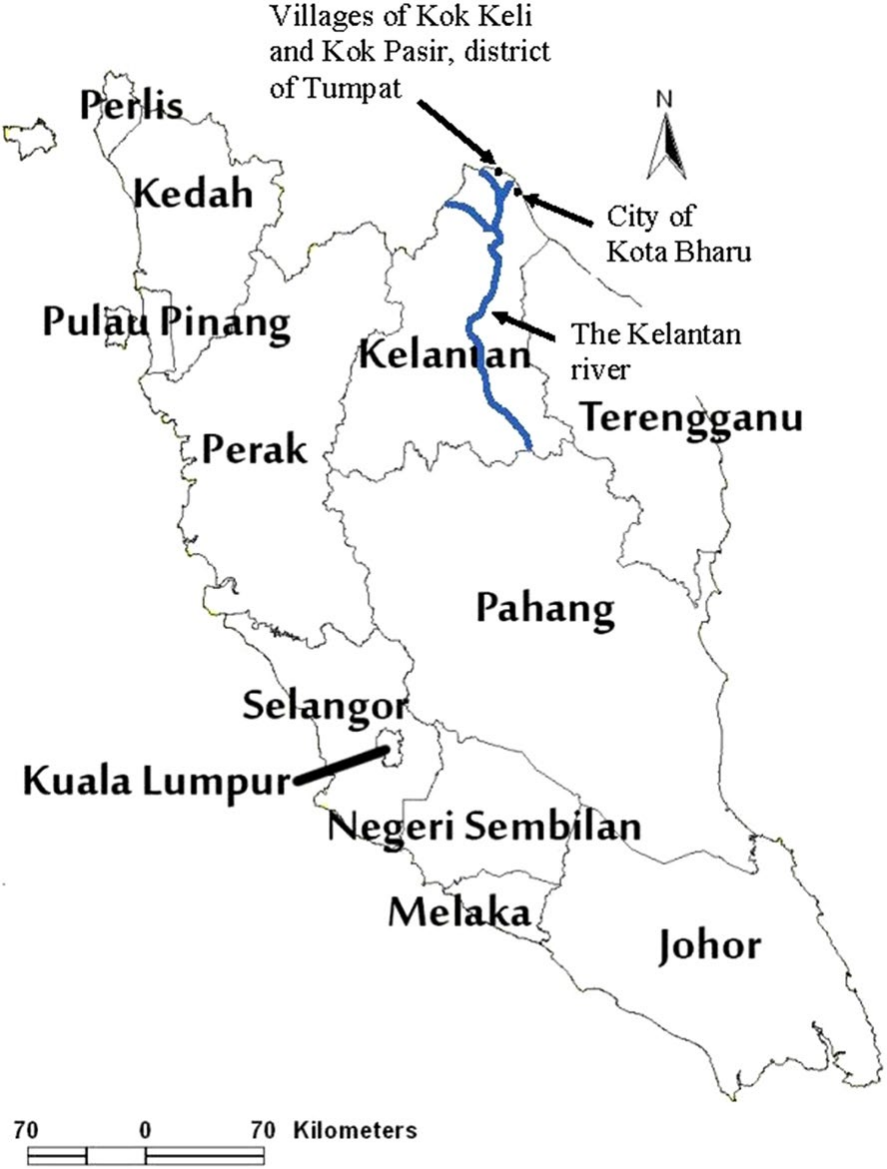
A map of Peninsular Malaysia showing the Kelantan river that caused the major flood, and location of the two villages that were involved in our study

In December 2014, a massive river-flood disaster affected 230,000 people in the north-eastern region of Peninsular Malaysia, leaving 2000 homeless and approximately 21 dead (Fig. 1). Many victims had poor water, sanitation and hygiene (WaSH) practices during the flood and post-flood period. They had limited access to clean water for drinking and preparing food as well as limited access to clean toilet facilities. It is postulated that ingestion of faecal pathogens in contaminated flood water because of poor WaSH practices may cause small intestinal bacterial overgrowth (SIBO) and dysbiosis in the gut [3]. As a result, adult flood victims may develop persistent abdominal pain akin to post-infectious irritable bowel syndrome (IBS) 3–12 months later [4, 5], impaired quality of life (QOL) and psychological well-being including anxiety and depression [6].

Our study objectives were to determine firstly the association between persistent abdominal pain and QOL, anxiety, depression and poor WaSH practices in a flood-affected community; secondly, to determine if SIBO was associated with abdominal pain in flood-affected adults with poor WaSH practices; and lastly, to describe the gut microbial taxa in stools of flood-affected adults. The current study may provide a direct example of how disturbances in the external environment (ecological niche) can result in a prolonged disorder of the homeostatic microbiome [5].

## Methods

### Study design and population

The present study was a cross-sectional study involving adult participants from two villages located approximately 25 km from the city of Kota Bharu, in the north-eastern region of Peninsular Malaysia (Fig. 1). The two villages, namely Kok Keli and Kok Pasir, have a combined population estimate of 3700 and both villages were badly affected during the flood in December 2014. A list of worst flood-affected households was provided by the community leaders and these households were approached sequentially between August 2015 and November 2015. Available individuals, one from each household, were consented and surveyed for symptoms, QOL, psychological disturbance and WaSH practices. In addition, participants were asked to provide breath samples for hydrogen breath testing and a stool sample for metagenomic analysis. Inclusion criteria consisted of participants aged ≥18 years (y) and affected by the river-flood in December 2014. Exclusion criteria included history of abdominal symptoms prior to the river-flood, history of antibiotics or probiotics for 3 months prior to and after flood, inability to provide breath and stool samples, chronic medical illnesses (especially neurological diseases e.g. strokes and cancers) and previous abdominal surgeries and psychiatric illnesses. This study was approved by the Universiti Sains Malaysia (USM) Human Research Ethics Committee (USM/JEPeM/15040133).

### Assessment of symptoms, quality of life and psychological distress

Participants were asked if they had new onset abdominal pain that persisted for 6 months after the flood. In order to exclude pre-existing functional gastrointestinal (GI) disorders, participants were asked for any prior history of abdominal symptoms (including dyspepsia, pain, loose stools, constipation etc.) and also whether they had taken any medications to relieve abdominal symptoms. Demographic data including age, gender, marital status and educational status were also captured. Questionnaires administered included the Malay version of Rome III Questionnaires for IBS, functional dyspepsia (FD) and also the gastroesophageal reflux disease questionnaire (GERDQ) [6–8]. A diagnosis of IBS and FD were made based on previously published Rome III criteria [9]. For QOL assessment, the validated Malay version of 36 items was administered; this questionnaire consists of four physical domains i.e. physical functioning (10-item), role limitations in physical health (4-item), bodily pain (2-item) and general health perception (5-item) and four mental domains i.e. social functioning (2-item), role limitations due to emotional problems (3-item), vitality or energy (4-item) and mental well-being (5-item) [10]. Each domain of SF-36 has a score range of 0–100, with lower score signifying worse QOL. For assessment of anxiety and depression, the Malay version of the Hospital Anxiety and Depression Scale (HADS) was administered; this questionnaire consists of 14 items with four-point Likert responses. Each domain of HADS is scored as a continuous variable [11].

### Assessment of water, sanitation and hygiene (WaSH) practices

According to WHO/UNICEF, poor water practice includes the use of water from contaminated sources; poor sanitation practice means no clean toilet facility; and poor hygiene practice includes washing hands with no soap, no hand-washing or no bathing facilities in the house [12]. Above is the basis for a specifically developed questionnaire to assess WaSH practices of victims during the flood. A group of experts (physicians and public health experts) was responsible to draft the 10-item questionnaire based on their WaSH experiences with flood victims but also with literature review. The WaSH practice questionnaire consists of three domains, namely water (4-item), sanitation (3-item) and hygiene (3-item). Responses were in 5-point Likert scale (Additional file 1). Scores for each domain and a mean total score of all domains of WaSH were calculated as continuous variables; higher scores signified poorer WaSH practices.

### Breath-testing for small intestinal bacterial overgrowth (SIBO)

SIBO is postulated to be associated with post-flood symptoms and hydrogen breath test is a suitable non-invasive method to diagnose SIBO. After an overnight fast, agreed participants would exhale end-expiratory breath samples into a collection bag at baseline. Then they were asked to drink 75 g of glucose in cold water [13]. At intervals of 15 min for the next 2 h, breath samples were collected and symptoms were recorded [13]. The breath samples were brought back to the hospital and tested within 24–48 h. A 40 mL of exhaled breath would be syringed into the machine (Quintron, Milwaukee, US) and levels of H_2_ and CH_4_ (in parts per million or ppm) determined. For a positive test, the following criteria were applied: a rise in H_2_ value (≥ 20 ppm) or CH_4_ values (≥ 10 ppm) above fasting baseline value or a sustained rise in H_2_ or CH_4_ of 5 ppm over three consecutive breath samples [13]. A rise in breath values as above and reproduction of symptoms were required to diagnose SIBO.

### Assessment of fecal specimen

Early morning fecal specimens defecated on a rice paper in lavatory bowl were collected in a clean plastic container. After that, two spatula portions of the fecal specimens were transferred into a sterile fecal collection tube, and capped tightly. The collection tube was pre-filled with 2–4 mL of RNAlater^®^ stabilization solution (Thermo Fisher Scientific, USA) and four glass beads [14]. The tube was shaken vigorously for 10 s to suspend the feces in the solution. Fecal samples were delivered to the laboratory within 24 h and then stored at − 20 °C. Total DNA from 20 mg of fecal samples, which were precipitated by centrifugation, was extracted using the QIAamp Fast DNA Stool Mini Kit (Qiagen, USA) according to the manufacturer’s instructions. Purified DNA was suspended in 2000 μL of Tris–EDTA buffer (pH 8.0). Polymerase chain reaction (PCR) amplification of the bacterial 16S rRNA gene V3–V4 region was performed with the TaKaRa Ex Taq HS Kit (TaKaRa Bio, Shiga, Japan) with the primer sets Tru357F (5′-CGCTCTTCCGATCTCTGTACGGRAGGCAGCAG-3′) and Tru806R (5′-CGCTCTTCCGATCTGACGGACTACHVGGGTWTCTAAT-3′) [15]. Each sample of DNA (1 µL) at 10–200 ng/µL was measured using a Nanodrop 2000 (Thermo Fisher Scientific, Waltham, MA, USA) according to the method by Odamaki et al. [15]. The samples of DNA were amplified in triplicate under the following conditions: preheating for 3 min at 94 °C followed by 20 cycles of denaturation for 30 s at 94 °C, annealing for 30 s at 50 °C, extension for 30 s at 72 °C and a final terminal extension for 10 min at 72 °C [15]. After that, the amplified DNA was verified based on the product size of PCR by QIAxcel system (Qiagen, Valencia, CA, USA). The combined PCR product was then amplified by the barcoded primers adapted for the Illumina MiSeq: Fwd 5′-AATGATACGGCGACCACCGAGATCTACACXXXXXXXXACACTCTTTCCCTACACGACGCTCTTCCGATCTCTG-3′ and Rev 5′-CAAGCAGAAGACGGCATACGAGATXXXXXXXXGTGACTGGAGTTCAGACGTGTGCTCTTCCGATCTGAC-3′, where X was labelled as a barcode base. The amplification of DNA was performed according to the method described above except that eight cycles were conducted. The second amplified DNA products were validated using QIAxcel system and purified by QIAquick 96 PCR Purification Kits (Qiagen, Valencia, CA, USA). The quantification of purified DNA products were then performed by Quant-iT PicoGreen dsDNA Assay Kits (Life Technologies, Carlsbad, CA, USA). After pooling the equal amounts of the amplicons from multiple samples, GeneRead Size Selection Kits (Qiagen, Valencia, CA, USA) were used to remove the primer dimers. An Illumina MiSeq instrument with a MiSeq v3 Reagent Kits (Illumina, Inc., San Diego, CA, USA) was used to sequence the pooled libraries. After the removal of sequences consistent with data from the Genome Reference Consortium human build 37 (GRCh37) or PhiX 174 from the raw Illumina paired-end reads, the 3′ region of each read with < 17 PHRED quality scores was trimmed. Trimmed reads < 150 bp in length with a mean quality score < 25 were also removed. The fastq-join script in EA-Utils (version 1.1.2-537) was used to combine the reads that passed the quality filters. For the taxonomic analysis, the sequences were analysed by the QIIME software package version 1.8.9 (http://qiime.org/). The potential chimeric sequences were removed by UCHIME, which was assigned to the open-reference operational taxonomic units (OTUs). The sequences were taxonomically classified by the Greengenes reference database [15].

### Statistical analysis

Continuous variables were presented in mean ± standard error of mean (SEM) unless otherwise mentioned. Analysis was performed using Chi square or Fisher-exact test for categorical data and t-test for continuous data. Binary logistic regression analysis (odds ratio [OR] and 95% confidence interval [CI]) was used to test for factors associated with abdominal pain, poor WaSH practices during flood and SIBO, respectively. Principal component analysis (PCoA) based on Jensen-Shannon divergence (JSD) was performed using R version 3.2.4 and linear discriminant analysis (LDA) effect size or LEfSe method for microbial taxa composition were performed on the Galaxy web site (https://huttenhower.sph.harvard.edu/galaxy) [16]. LDA effect size or LEfSe provides an estimate and ranking of differentially abundant microbial taxa in the faecal sample [15]. A *P*-value < 0.05 was considered as significant. Bonferroni correction was applied to each domain of the WaSH practice and SF-36 questionnaires.

## Results

### Characteristics of study participants

**Fig. 2:**
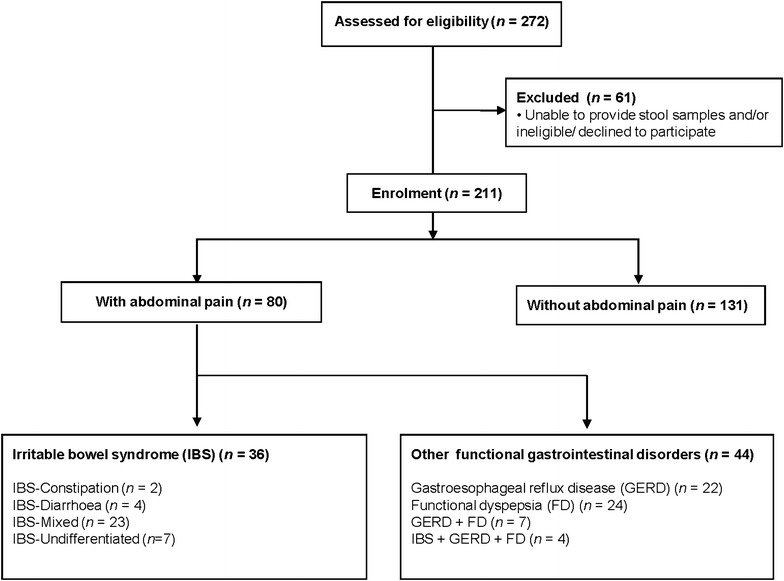
Flow chart of study recruitment

Individuals from 272 affected households were screened and 211 eligible participants (mean age 54.5 ± 1.0 years, age range 19–86 years, females 71%) met the study criteria and agreed to participate (Fig. 2). Characteristics of participants are shown in Table 1. Abdominal pain that persisted following flood was seen in 37.9% (*n* = 80). Of those with abdominal pain, 45% (*n* = 36) had IBS. Among the IBS participants, 75% (*n* = 27) were diarrhoea and/or mixed-subtype, 5.6% (*n* = 2) were constipation-subtype and 19.4% (*n* = 7) were undifferentiated-subtype. In addition, of those with abdominal pain but did not have IBS (55% or *n* = 44), these participants had functional dyspepsia (FD) (30% or *n* = 24), GERD (27.5% or *n* = 22) and overlap between FD and GERD (15.9% or *n* = 7). Overlap of all three conditions in participants with abdominal pain i.e. FD, GERD and IBS was present in 9.1% (n = 4). None of those without abdominal pain (*n* = 131) had any of the above functional GI disorders after flood.

**Table 1:** Factors associated with persistent abdominal pain in a flood-affected community

	With abdominal pain (*n* = 80)	Without abdominal pain (*n* = 131)	*P* value
Age, mean (SEM), years	52.0 (1.9)	56.0 (1.2)	0.07
Gender, female, *n* (%)	34 (79.1)	64 (69.6)	0.25
Education (primary and beyond), *n* (%)	69 (86.3)	111 (84.7)	0.8
Married, *n* (%)	75 (93.8)	128 (97.7)	0.1
WaSH (during flood), total score, mean (SEM)	19.6 (0.7)	18.1 (0.4)	0.04^#^
WaSH (during flood), water, mean (SEM)	8.8 (0.5)	7.4 (0.2)	0.005^#^
WaSH (during flood), sanitation, mean (SEM)	6.0 (0.2)	5.9 (0.2)	0.8
WaSH (during flood), hygiene, mean (SEM)	4.9 (0.2)	4.8 (0.2)	0.9
SF-36, total score, mean (SEM)	69.8 (2.1)	76.3 (1.8)	0.02^#^
Physical functioning, mean (SEM)	66.0 (3.2)	68.4 (2.5)	0.6
Physical health, mean (SEM)	67.2 (4.6)	70.1 (3.4)	0.6
Emotional problem, mean (SEM)	70.5 (4.7)	91.0 (7.9)	0.04^#^
Energy, mean (SEM)	68.3 (2.0)	71.2 (1.7)	0.3
Mental well-being, mean (SEM)	74.6 (1.8)	77.9 (1.5)	0.2
Social functioning, mean (SEM)	77.9 (2.4)	83.5 (2.0)	0.07
Bodily pain, mean (SEM)	72.4 (2.7)	80.9 (1.9)	0.009^#^
General health perception, mean (SEM)	63.2 (1.9)	64.4 (1.7)	0.7
Anxiety, mean (SEM)	4.0 (0.4)	2.9 (0.4)	0.04^#^
Depression, mean (SEM)	3.8 (0.4)	3.5 (0.3)	0.6
GERDQ score, mean (SEM)	6.8 (0.2)	6.2 (0.1)	0.008^#^
Functional dyspepsia (FD), *n* (%)	24 (33.8)	10 (9.3)	< 0.001^#^
Functional constipation (FC), *n* (%)	34 (44.2)	32 (27.8)	0.02^#^

*SEM* standard error of the mean, *WaSH* water, sanitation and hygiene

^#^Significant *P* value < 0.05

### Relationship between abdominal pain and IBS with WaSH practices, QOL and psychological morbidity

Relationship between abdominal pain and IBS with QOL, psychological co-morbidity and WaSH practices is shown in Table 1. With regards to WaSH practices among all flood victims, the mean total WaSH score was 18.7 ± 0.4 and hygiene scored the lowest at 4.8 ± 0.1. Participants with vs. without abdominal pain were significantly associated with increased mean total WaSH score (19.6 ± 0.7 vs. 18.1 ± 0.4, *P* = 0.04). Likewise, participants with vs. without IBS had a higher mean total WaSH score (21.0 ± 1.2 vs. 18.4 ± 0.4, *P* = 0.006). Among the three domains, only poor water practices (including poor quality, colour and taste of water supply during flood) were significantly associated with vs. without abdominal pain (8.8 ± 0.5 vs. 7.4 ± 0.2, *P* = 0.005).

Among all flood victims included in the study, the mean total score of SF-36 was 73.8 ± 1.4 and the lowest scores were general health (64.0 ± 1.3) and energy (70.1 ± 1.3). Participants with vs. without abdominal pain had a significantly lower bodily pain score (72.4 ± 2.7 vs. 80.9 ± 1.9, *P* = 0.009) and likewise, with vs. without IBS (65.4 ± 4.1 vs. 80.0 ± 1.8, *P* = 0.001). In contrast to abdominal pain, those with vs. without IBS also reported lower scores for mental well-being (68.8 ± 2.5 vs. 77.1 ± 1.4, *P* = 0.007) and social functioning (71.9 ± 3.5 vs. 83.0 ± 1.9, *P* = 0.007).

The mean anxiety score of all flood victims in study was 3.4 ± 0.3 and their mean depression score was 3.6 ± 0.2. Participants with vs. without abdominal pain had significantly higher anxiety scores (4.0 ± 0.4 vs. 2.9 ± 0.4, *P* = 0.04), and likewise, with vs. without IBS (5.3 ± 0.5 vs. 3.1 ± 0.3, *P* = 0.001). No significant difference in depression scores was observed between participants with vs. without abdominal pain (*P* = 0.6) and likewise, with vs. without IBS (*P* = 0.06).

### SIBO is associated with worse WaSH, QOL and anxiety but not pain or IBS post-flood

Of 211 participants that completed the questionnaires, 135 consented for subsequent breath testing for SIBO. Results of these 135 participants were subsequently analysed and reported for association between SIBO and pain. Of the 135 participants (mean age 55.6 ± 1.3 years, females 98 or 72.6%), 12.6% (*n* = 17) were SIBO positive. Of those positive for SIBO, 35.3% (*n* = 6) had abdominal pain and 29.4% (*n* = 5) had IBS. Frequency of participants positive for SIBO was not statistically different between those with vs. without abdominal pain (*n* = 6 vs. 11, *P* = 0.7) and likewise, with vs. without IBS (*n* = 5 vs. 12, *P* = 0.6). Although not associated with pain or IBS, those with vs. without SIBO reported worse water practices during flood (9.5 ± 2.0 vs. 7.8 ± 0.2, *P* = 0.04), lower physical functioning (51.2 ± 8.3 vs. 67.8 ± 2.5, *P* = 0.02), lower social functioning (70.7 ± 6.8 vs. 84.0 ± 1.7, *P* = 0.01) and higher anxiety scores (5.2 ± 1.3 vs. 3.1 ± 0.2, *P* = 0.01).

### Gut dysbiosis is associated with psychological disturbance and abdominal pain

**Fig. 3:**
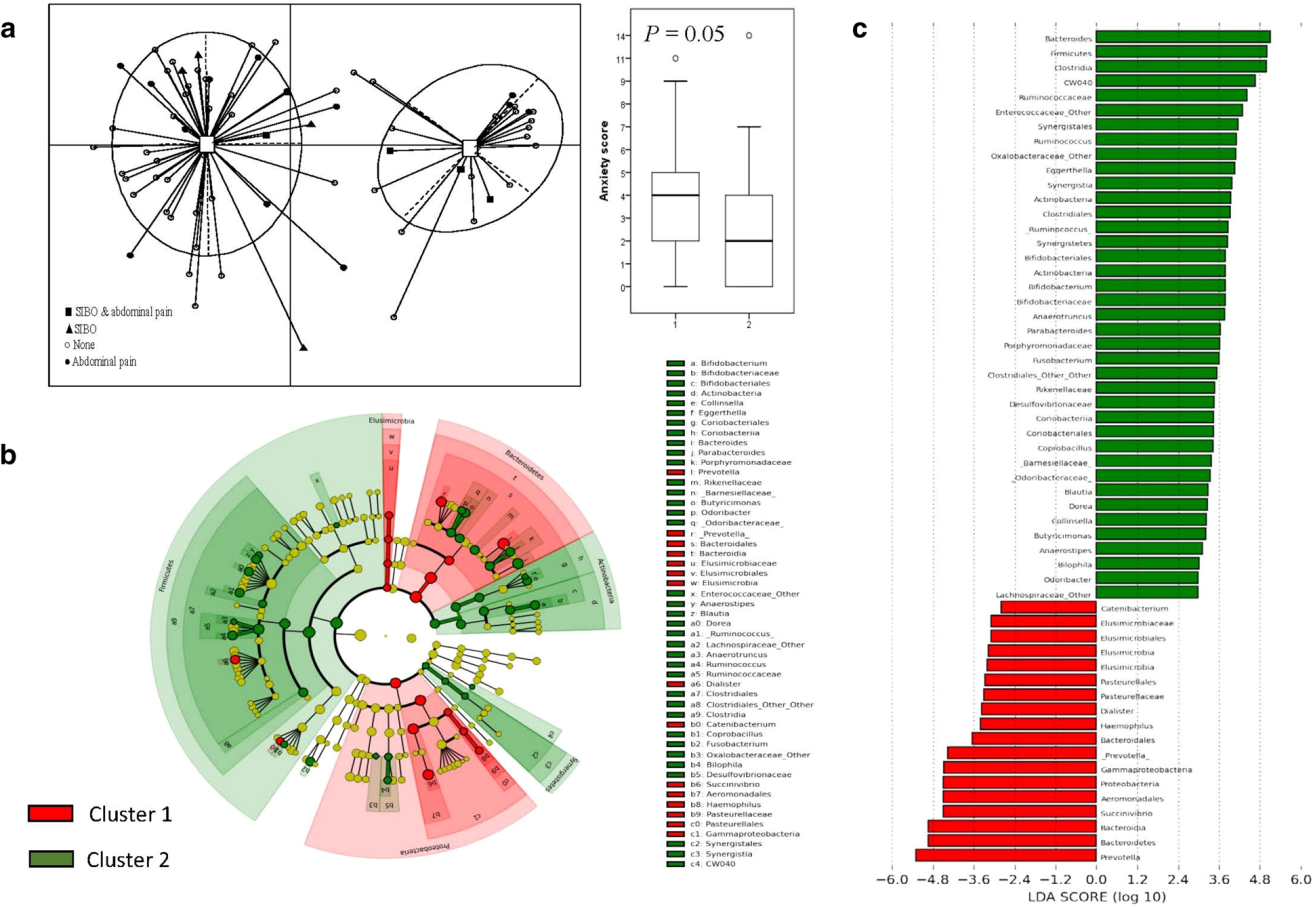
Principal component analysis (PCoA) based on Jensen-Shannon divergence identifies two clusters based on anxiety scores (**a**), and the score is higher in cluster 1 than 2. Not shown here is the Shannon Index which is significantly lower in cluster 1 than 2 (4.8 vs. 5.5, *P* < 0.001). Taxonomic representation of microbial composition of both clusters is shown in (**b**), with cluster 1 in red and cluster 2 in green. Histogram of the LDA effect size for both clusters is shown in (**c**). The most differentially abundant bacterial taxa observed in cluster 1 are the phyla Bacteroidetes (including the genus *Prevotella*) and Proteobacteria with effect size of 4.8

**Fig. 4:**
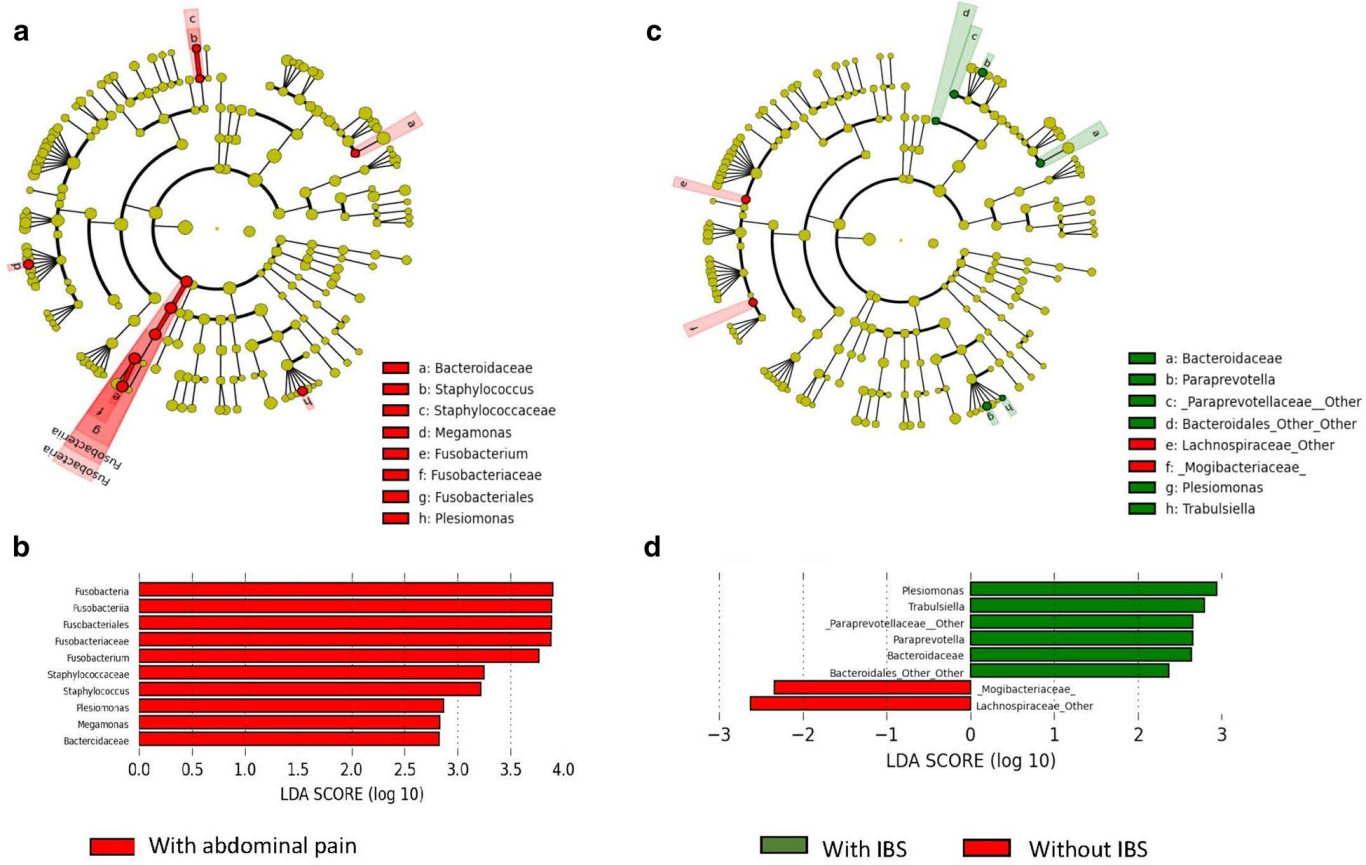
Taxonomic representation and histogram of the LDA effect size for microbial composition in those with persistent abdominal pain (**a** and **b**) and IBS (**c** and **d**) are shown here. For those victims with abdominal pain (**a** and **b**), Fusobacteria is the most abundant with effect size of 4.0. Others of significance include the *Staphylococcus, Megamonas* and *Plesiomonas*. For those with IBS (**c** and **d**), *Plesiomonas* and *Trabulsiella* are the most abundant with effect size approaching 3.0

Of 135 participants consented for breath testing, 73 agreed to give their stools for high throughput sequencing and subsequent metagenomic analysis. Of 73 participants (mean age 55.8 ± 1.6 years, females 53 or 72.6%), 21.9% had abdominal pain and 17.8% had IBS. The predominant phyla in all 73 participants were Bacteroidetes (37.1%), Firmicutes (24.6%) and Proteobacteria (8.4%). With PCoA of gut microbiota composition, two different clusters were observed for anxiety (*P* < 0.05) but not other scores (Fig. 3). The most differentially abundant bacterial taxa observed in the cluster with a higher anxiety score (mean score 4.0, cluster 1) were the phyla, Bacteroidetes (including the genus Prevotella) and Proteobacteria with effect size of 4.8. The Shannon Index was significantly lower in the cluster with more anxiety (mean score 4.0, cluster 1) than with less anxiety (mean score 2.0, cluster 2) (4.8 vs. 5.5, *P* < 0.001). Figure 4a, b shows the taxonomic representation and histogram of LDA scores of participants with abdominal pain. Among those with abdominal pain, the phylum Fusobacteria was the most abundant with LDA effect size of 4.0 and other abundant organisms included the genus *Staphylococcus, Megamonas* and *Plesiomonas*. Figure 4c, d shows the taxonomic representation and histogram of LDA scores of participants with and without IBS. The most differentially abundant bacteria taxa observed in those with IBS was the genus *Plesiomonas* and *Trabulsiella* with effect size approaching 3.0.

## Discussion

Our study provides evidence that persistent abdominal pain is common (37.9% of studied population) over 6 months following a flood disaster, and almost half of the flood affected adults with abdominal pain also fulfilled the Rome III criteria for IBS. Flood-affected adults with abdominal pain due to IBS reported a higher IBS severity score and two-thirds were of diarrheal and/or mixed-type. For comparison, 36% of IBS cases were reported over 24 months in the Walkerton outbreak of *Escherichia coli* and *Campylobacter jejuni* found in contaminated water, and 60.7% reported watery stools in at least 25% of the time [17]. Because of similar pathophysiology affecting the brain-gut axis, other functional GI disorders including FD and GERD were reported among flood victims who developed abdominal pain which was not related to IBS, and they also overlapped with IBS participants [6, 18].

Patients with sporadic IBS have a poorer health-related QOL [19–21] and the same may occur in flood victims who developed IBS in our study. SF-36 allows us to assess both physical and mental functioning of these victims. Bodily pain was found to be significantly associated with abdominal pain after flood (Table 1) and bodily pain in SF-36 may be conceptually related to abdominal pain and hence explain the significant association observed between the two. Flood victims with abdominal pain and IBS were also more anxious and this is in keeping with previous reports of anxiety in IBS [19–21].

Previous published studies have shown reduction in QOL among flood victims in both physical and mental domains, for example, poor general health and energy in a Chinese study and poor physical and social functioning in a Korean study [22, 23]. Similarly, QOL was most reduced especially in physical domain of general health but less so of mental domain of energy among flood victims in our study. In contrast, from previous published studies, IBS patients experience decrements in QOL most pronounced in energy and bodily pain but significantly less in physical functioning [24, 25]. Our results are in agreement with previous studies where mental domain in flood victims with IBS is most affected, especially mental functioning and social functioning [24, 25]. This is also in agreement with the higher anxiety score that we observed in flood victims with IBS and not in those without IBS.

Our study provides evidence that persistent abdominal pain following massive flood is associated with poor WaSH practices. It is not known how poor WaSH practices can cause abdominal pain but poor WasH practices may be associated with fecal contamination of water. Hence, our study postulated that SIBO may be increased following flood because of poor WaSH practices, and we indeed showed the positive association between the two, and in addition, SIBO results in worse QOL although not abdominal pain or IBS. It has been shown that adults and children who inhabit a fecally contaminated environment are at risk for developing environmental enteropathy (EE) and/or tropical sprue later in life, both are forms of small intestinal bacterial overgrowth [26]. Particularly, poor sanitation due to absence of clean toilet facilities during flood is associated with fecal contamination, and half of our study participants reported poor sanitation. Many toilets were unusable during and after the flood, being submerged in the flood water and mud, and therefore victims had no means for clean sanitation and hygiene.

It is unknown if anxiety or environmental microbiota is the inciting factor for abdominal pain or IBS after flood, but in the post-infectious IBS model, the inciting factor is microbiota [27], and it then affects anxiety through the gut-brain axis. We observed that microbial abundance seems to shift towards the phyla Bacteroidetes and Proteobacteria in anxious flood victims (Fig. 3). Abundance of these organisms has been associated with inflammatory bowel disease, in particular the expansion of Proteobacteria is considered as a significant marker of gut dysbiosis [28, 29]. In addition, Fusobacteria were found to be more abundant in our flood victims with abdominal pain (Fig. 4). Fusobacteria are anaerobic gram-negative bacilli that have been implicated in acute appendicitis, inflammatory bowel disease and SIBO [30–32]. *Staphylococcus, Megamonas* and *Plesiomonas* were also implicated with abdominal pain in our study and these organisms are likely environmentally derived pathogens from contaminated flood water [33]. However, it is also possible that these are pre-existing pathobionts that have been expanded following flood-related gastroenteritis [33]. Likewise, in those flood victims with IBS, there were more *Plesiomonas* and *Trabulsiella* in their stools. The above findings suggest that a significant cause of abdominal pain among flood victims is related to gut dysbiosis and the dysbiosis is likely of environmental origin because of exposure to contaminated flood water [33]. Based on the above findings, it is possible that to manipulate or restore the microbial homeostasis among flood victims with the use of probiotics. From IBS-based studies, besides improving visceral sensitivity, probiotics can protect colonic epithelial cells from invasive environmental micro-organisms [6]. Additionally, probiotics can improve QOL, anxiety and depression mediated through their central effects on the gut-brain axis. Further studies are needed before probiotics can be recommended for post-flood abdominal symptoms.

There are a few limitations to our study. There were more participants in their fifties and also females because many young adults move away from villages after floods. Another limitation is the recall bias of WaSH practices during flood and this was reflected indirectly by the relatively low odds ratio in comparison to the risk of IBS. Although our study did not test the reliability of the WasH questionnaire, the low scores of WaSH practices in our study were consistent with poor hygiene practice and sanitation facilities in the population [34–36]. Likewise, the exclusion of participants with a previous antibiotics and probiotics might introduce bias in the study towards a falsely small percentage of abdominal pain in the population. It is possible for some victims to have pre-existing functional disorders but these disorders were screened negative using questionnaires. Furthermore, participants did not report any prior treatments for any abdominal symptoms before study recruitment. In addition, only anxiety and depression were evaluated, not the full spectrum of psychological disturbances. Our study did not find an association between SIBO and abdominal pain or IBS because of small sample size and because of method of testing (glucose rather than lactulose). The rates of abdominal complaints in our study were higher than previously reported [37] but studied populations are different. Finally, our study did not perform endoscopy or biopsy from participants with abdominal pain because of logistic issues.

## Conclusion

This study suggests that gut dysbiosis because of environmentally derived organisms following poor WaSH practices may explain the persistent abdominal pain and IBS that developed after a major environmental disaster. Probiotics may be an attractive option for post-flood abdominal symptoms but further research is needed.

## Additional file

**Additional file 1.** The Water, Sanitation and Hygiene (WaSH) Practices Questionnaire.
