# Supplementary material for: Exposure to environmental microbiota explains persistent abdominal pain and irritable bowel syndrome after a major flood
Source: Gut Pathog. 2017 Dec 14;9:75. doi: 10.1186/s13099-017-0224-7 (PMC5729606; doi:10.1186/s13099-017-0224-7)
Supplement: Supplementary file 1 — Additional file 1. The Water, Sanitation and Hygiene (WaSH) Practices Questionnaire. [file 13099_2017_224_MOESM1_ESM.docx]

**Additional file 1**

**The Water, Sanitation and Hygiene (WaSH) Practices Questionnaire**

Please based the answers in each of the following section on your experience 6 months ago immediately after the massive flood last year (December 2014)

| Section A: Household Water Practices |
| --- |

Please (√) your answer on the scale of 1 to 5 as per follow:

| 1. Overall, how would you grade the **quality/ cleanliness** of your water supply? | 1  Very good | 2  Good | 3  Less good | 4  Poor | 5  Very poor |
| --- | --- | --- | --- | --- | --- |

| 1. Most of the time, how would you grade the **colour** of your household major water supply? | 1  Very clear | 2  Clear | 3  Less clear | 4  Dark | 5  Very dark |
| --- | --- | --- | --- | --- | --- |

| 1. Most of the time, how would you grade the **taste** of your household major water supply ? | 1  Very good | 2  Good | 3  Less good | 4  Poor | 5  Very poor |
| --- | --- | --- | --- | --- | --- |

| 1. If given a choice, would you use the water supply for drinking/ cooking purpose? | 1  Without reservation | 2  Little  reservation | 3  A lot of reservation | 4  Only used if no other option | 5  Will not use at all |
| --- | --- | --- | --- | --- | --- |

| Section B: Sanitation Practices |
| --- |

Please (√) your answer on the scale of 1 to 5 as per follow:


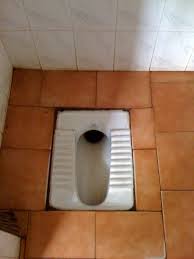

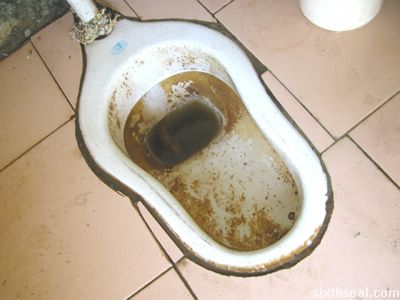


| 1. How would you grade the condition of the facilities used for defecation/ urination | 1  Very clean | 2  Clean | 3  Less clean | 4  Dirty | 5  Very dirty |
| --- | --- | --- | --- | --- | --- |

| 1. How would you grade the area to dispose the household waste/ garbage? | 1  Very clean | 2  Clean | 3  Less clean | 4  Dirty | 5  Very dirty |
| --- | --- | --- | --- | --- | --- |

| 1. If given a choice, would you use the above facilities for sanitation purpose? | 1  Without reservation | 2  Little reservation | 3  A lot of reservation | 4  Only used if no other option | 5  Will not use at all |
| --- | --- | --- | --- | --- | --- |

| Section C: Hygiene Practices |
| --- |

Please (√) your answer on the scale of 1 to 5 as per follow:

| 1. Overall, how would you grade your household hygiene practice? | 1  Very good | 2  Good | 3  Less good | 4  Poor | 5  Very poor |
| --- | --- | --- | --- | --- | --- |

| 1. How frequently do you practice hand-washing for example after latrine use, after handling rubbish, or before food preparation? | 1  All the times | 2  Most of the time | 3  Some of the time | 4  Very infrequent | 5  Never |
| --- | --- | --- | --- | --- | --- |

| 1. How frequent do you boil your drinking water? | 1  All the time | 2  Most of the time | 3  Some of the time | 4  Very infrequent | 5  Never |
| --- | --- | --- | --- | --- | --- |
